# ERPs responses to dominance features from human faces

**DOI:** 10.1038/s41598-022-25370-4

**Published:** 2022-12-02

**Authors:** Chengguo Miao, Xiaojun Li, Edmund Derrington, Frédéric Moisan, Yansong Li, Jean-Claude Dreher

**Affiliations:** 1 Reward, Competition and Social Neuroscience Lab, Department of Psychology, School of Social and Behavioral Sciences, Nanjing University, Nanjing, 210023 China; 2 School of Teacher Education, NanJing XiaoZhuang University, Nanjing, China; 3 Neuroeconomics, Reward and Decision-Making Team, Institut des Sciences Cognitives Marc Jeannerod, Centre National de la Recherche Scientifique, 67 bd Pinel, Bron, 69675 France; 4 Emlyon Business School and GATE CNRS, UMR 5824, 23 Av. Guy de Collongue, Ecully, 69130, France; 5 Institute for Brain Sciences, Nanjing University, Nanjing, China

**Keywords:** Social neuroscience, Human behaviour

## Abstract

Social dominance is an important feature of social life. Dominance has been proposed to be one of two trait dimensions underpinning social judgments of human faces. Yet, the neural bases of the ability to identify different dominance levels in others based on intrinsically facial cues remains poorly understood. Here, we used event-related potentials to determine the temporal dynamics of facial dominance evaluation based on facial features signaling physical strength/weakness in humans. Twenty-seven participants performed a dominance perception task where they passively viewed faces with different dominance levels. Dominance levels did not modulate an early component of face processing, known as the N170 component, but did modulate the late positive potential (LPP) component. These findings indicate that participants inferred dominance levels at a late stage of face evaluation. Furthermore, the highest level of dominant faces and the lowest level of submissive faces both elicited higher LPP amplitudes than faces with a neutral dominance level. Taken together, the present study provides new insights regarding the dynamics of the neurocognitive processes underlying facial dominance evaluation.

## Introduction

Social hierarchy is an essential and pervasive feature of groups living in many species, including humans, where hierarchical differentiation has a significant influence on behavior, motivation, and health^1,2^. High-ranking animals tend to have more access to food resources and territory, and also have a higher chance of reproductive success^3^. In humans, assessing the relative rank of conspecifics is crucial to successfully navigating our complex social environments. One fundamental distinction concerning social hierarchy representations is that they can be assessed according to dominance cues e.g., facial features, physical attributes such as body size, posture, and aggressive expressions), to rapidly evaluate the strength of potential competitors, and to avoid costly physical conflict^4^. Dominance hierarchies can also be learned by observation or through direct competitive dyadic interactions against rivals^5^. Each of these processes has been shown to engage specific brain networks^6,7^.

Here, we focus on the ability to assess socially dominant individuals based on facial features, which is an essential skill to avoid costly competition leading to social defeats^8^. Dominance is one of two trait dimensions underpinning social judgments of human faces^9–12^. From an evolutionary perspective, identifying the dominance status from features of faces is important for reproductive success. For example, a number of studies have found associations between adult men’s facial width-to-height ratio (fWHR) and perceived likelihood of dominance and aggression^13,14^. For this reason, increasing efforts have been devoted to understanding how individuals recognize or infer dominance hierarchies based on facial dominance evaluation in humans^6^. Recent progress in social neuroscience research has advanced our understanding of this matter by delineating the neurocognitive subprocesses of facial dominance evaluation^15^.

Within this domain, two ERP components, which have traditionally been found to be modulated by emotional face processing that is usually indexed by significantly increased amplitudes for emotional faces over neural faces^16,17^, may also be sensitive to dominance hierarchy during face processing, namely the late positive potential (LPP) and the N170^6^. The LPP refers to a positive-going wave, peaking between approximately 400 and 700 ms following face presentation, with maximum amplitude over the parietal scalp region. This component has been consistently observed during the social categorization of faces (familiarity, race, and gender). The LPP elicited by familiar faces is thought to reflect semantic and personal identity representations^18–20^, while those evoked by gender and race have been considered to reflect the cognitive assessment of face and category-related information^21,22^ or to reflect the manifestation of social evaluations^23^. Drawing on this literature, it is rational to speculate that the LPP would be modulated by a major feature of social categorization, namely the hierarchical dominance status, during face processing. Indeed, several electrophysiological studies have consistently found modulation of the LPP during facial dominance evaluation^24–29^. Since the LPP has been interpreted to reflect evaluations of faces on social dimensions during face processing, these results suggest that the inference of dominance hierarchies occurs during the late stage of face processing. In parallel, the N170 is a negative-going wave, peaking between 140 and 170 ms following the presentation of the face, with its maximum amplitude at the occipitotemporal sites. This component is thought to reflect the structural encoding of faces that occurs during an early stage of face processing^30^. Unlike the LPP, empirical research on whether social categories modulate the N170 has produced more conflicting findings^30^. Similarly, with regard to whether facial dominance modulates the early stage of face processing as indexed by the face-sensitive N170 component, evidence concerning this problem remains limited and conflicting. Specifically, modulation of the N170 by facial dominance has consistently been reported in EEG studies where facial dominance was conveyed through facial expression^31^ or simultaneous presentation of dominance-conveying symbols (different number of stars) with emotionally neutral faces^26,27^. However, this was not the case in studies where facial dominance was learned either via the direct experience of competitive interactions^24^, or when occupational labels were presented immediately before the faces^25^. This discrepancy has been argued to result from methodological differences in manipulating facial dominance^25^. Specifically, in the second line of research, facial dominance was manipulated through either learning from the outcome of direct interactions or associating professional dominance with the faces. Thus, the inference of facial dominance would not rely on perceptual identification of explicit dominance-conveying cues, but rather the memory of dominance hierarchies established during the learning phase, or the knowledge of professional dominance. Consequently, participants’ facial dominance evaluation would occur during the late phase of face processing, usually reflected by the increased amplitude of the LPP. Previous EEG findings that identified the dynamic processing of neurocognitive subprocesses of facial dominance evaluation have been limited by the fact that they did not manipulate facial dominance by varying intrinsic dominance-related facial features. The use of dominance-conveying symbols (e.g., stars) that are extrinsically linked to the faces as dominance cues has been argued to result in a potential interference of the status symbols with the evaluation of facial dominance, thereby introducing a potential confounding effect on participants’ inference of dominance hierarchies during facial dominance evaluation. As a consequence, manipulating facial dominance by varying only intrinsic dominance-related facial features provides a solution to the aforementioned issue. By doing so we can achieve an unambiguous examination of the neurocognitive subprocesses of facial dominance evaluation. For this reason, the nature of neurocognitive subprocesses associated with facial dominance evaluation still needs to be elucidated.

The present ERPs study was designed to improve our understanding of this issue. Since facial dominance judgments have been revealed to be sensitive to facial masculinity and maturity cues signaling physical strength/weakness of the person^10,32^, we manipulated facial dominance parametrically by only varying these dominance-related facial cues to control for the confounding effect described above. Faces that varied on the dominance dimension in terms of facial masculinity and maturity cues that signal physical strength/weakness were taken from a validated, computer-generated face database^33^. This database contains 25 different faces that can each be morphed to different dominance levels along a dominance scale ranging from − 3 (most submissive) to 0 (neutral) to + 3 (most dominant). We developed a dominance perception task in which participants passively viewed these faces. To confirm that participants were sensitive to different levels of facial dominance, passive trials were randomly interleaved with active trials where participants had to identify which one of two faces, of a single identity, was more dominant. Drawing on recent ERP findings^24–27,29,31^, we focused on two ERP components related to facial dominance processing, the N170 and the LPP. These two ERP components can help to explore how facial dominance related to physical strength/weakness exerts a temporally dynamic influence on face processing. Specifically, given that there is increasing evidence that dominance hierarchy does not modulate the early step of face processing^25,34^, we can expect that facial dominance levels would not modulate the N170 component during face processing in our study. In contrast, considering that the literature available has consistently reported that dominance hierarchy modulates the late step of face processing^24–26,29^, we expect that dominant faces elicited higher LPP amplitudes than faces with a neutral dominance level during face processing.

## Results

### Behavioral results

**Figure 1:**
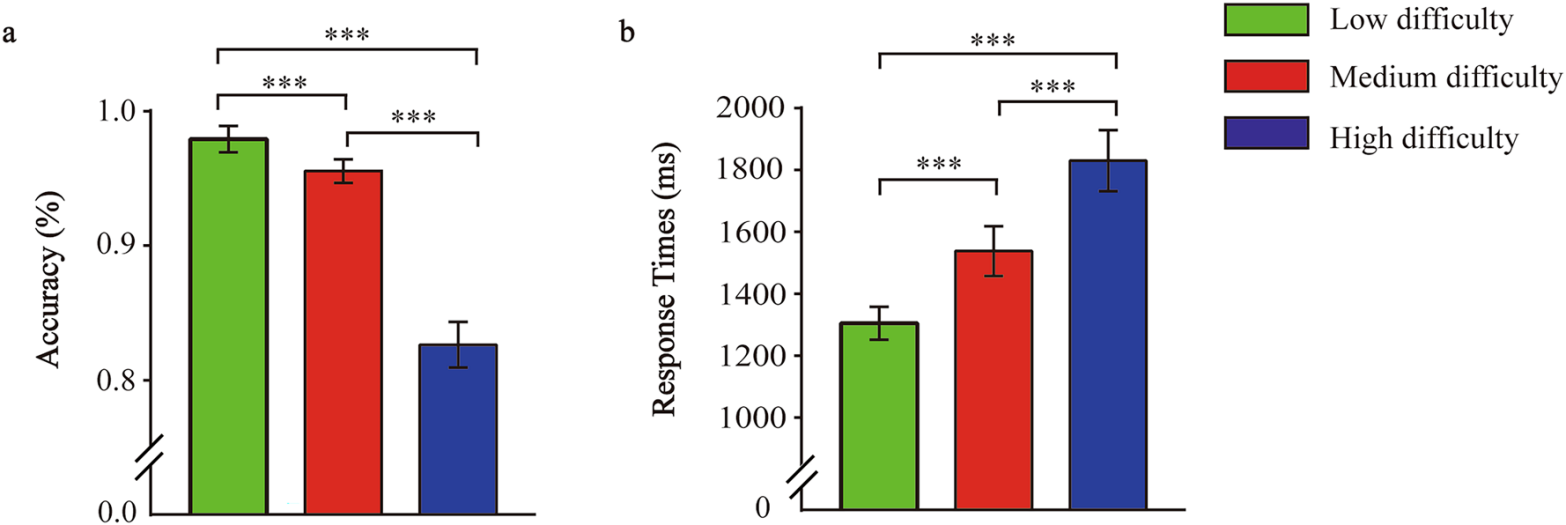
Performance during the active trials. (**A**) Bar plots of accuracy according to the level of difficulty. (**B**) Bar plots of reaction times according to the level of difficulty. Participants were indeed able to discriminate dominant from submissive faces (chance level of accuracy 50%). Error bars indicate standard error of mean (SE), (*p < 0.05, **p < 0.01, ***p < 0.001).

We used active trials to maintain attention to relevant stimulus features and to confirm that participants were indeed sensitive to different dominance levels. With regard to accuracy, a one-way ANOVA revealed a significant effect of difficulty (*F*(1, 26) = 97.618, *p* < 0.001, *η*^2^_*p*_ = 0.790) (Fig. 1A). A post hoc test revealed that accuracy was significantly higher for low difficulty trials (*M* = 0.98, *SE* = 0.01) than for both medium trials (*M* = 0.96, *SE* = 0.01; t = 3.184, *p* = 0.004) and high difficulty trials (*M* = 0.83, *SE* = 0.02; t = 11.555, *p* < 0.001). Furthermore, accuracy was also significantly higher for medium trials than for high difficulty trials (*p* < 0.001). Regarding the RTs, the one-way ANOVA revealed a significant effect of difficulty (*F*(1, 26) = 61.434, *p* < 0.001, *η*^2^_*p*_ = 0.703) (Fig. 1B). Post-hoc test revealed that RTs were significantly faster for low difficulty trials (*M* = 1305.29 ms, *SE* = 53.31) than for both medium difficulty trials (*M* = 1538.01 ms, *SE* = 79.90; t = 6.612, *p* < 0.001) and high difficulty trials (*M* = 1830.24 ms, *SE* = 98.32; t = 8.554, *p* < 0.001), and RTs were also significantly faster for medium difficulty trials than for high difficulty trials (t = 6.981, *p* < 0.001).

### Electrophysiological data

#### N170

**Figure 2:**
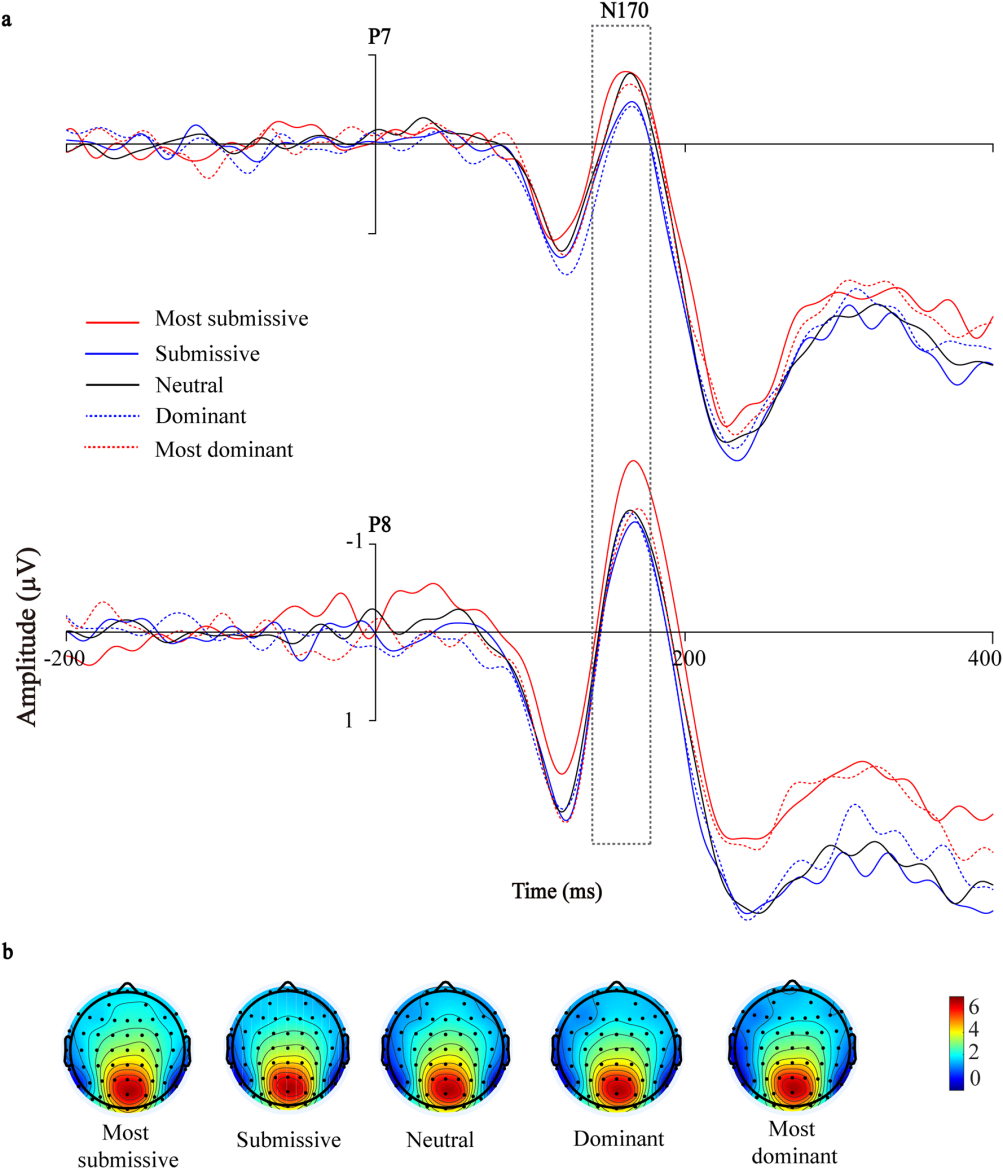
The N170 related to facial dominance evaluation. (**A**) The N170 over the temporo-parietal region (P7 and P8) as a function of dominance levels (− 3 SD, − 2 SD, 0 SD, + 2 SD, + 3 SD). (**B**) Topographical voltage distributions within 140–180 ms centered on the peak of N170 elicited by faces with different levels of dominance. Positive isopotential lines are in red and negative isopotential lines are in blue.

The N170 had a mean peak latency of 159.76 ms (*SE* = 0.82) after face onset. Our repeated-measures ANOVA on N170 latency failed to find a significant main effect of dominance levels (*F*(1, 26) = 1.933, *p* = 0.110, *η*^2^_*p*_ = 0.069) or electrodes (*F*(1, 26) = 0.438, *p* = 0.514, *η*^2^_*p*_ = 0.017). Moreover, there was no significant interaction between them (*F* (1, 26) = 2.499, *p* = 0.067, *η*^2^_*p*_ = 0.088). Likewise, regarding the N170 amplitude, we failed to find a significant main effect of dominance levels (*F*(1, 26) = 1.940, *p* = 0.109, *η*^2^_*p*_ = 0.069) or electrodes (*F*(1, 26) = 0.898, *p* = 0.352, *η*^2^_*p*_ = 0.033) and there was no significant interaction (*F*(1, 26) = 0.567, *p* = 0.687, *η*^2^_*p*_ = 0.021) (Fig. 2).

#### LPP

**Figure 3:**
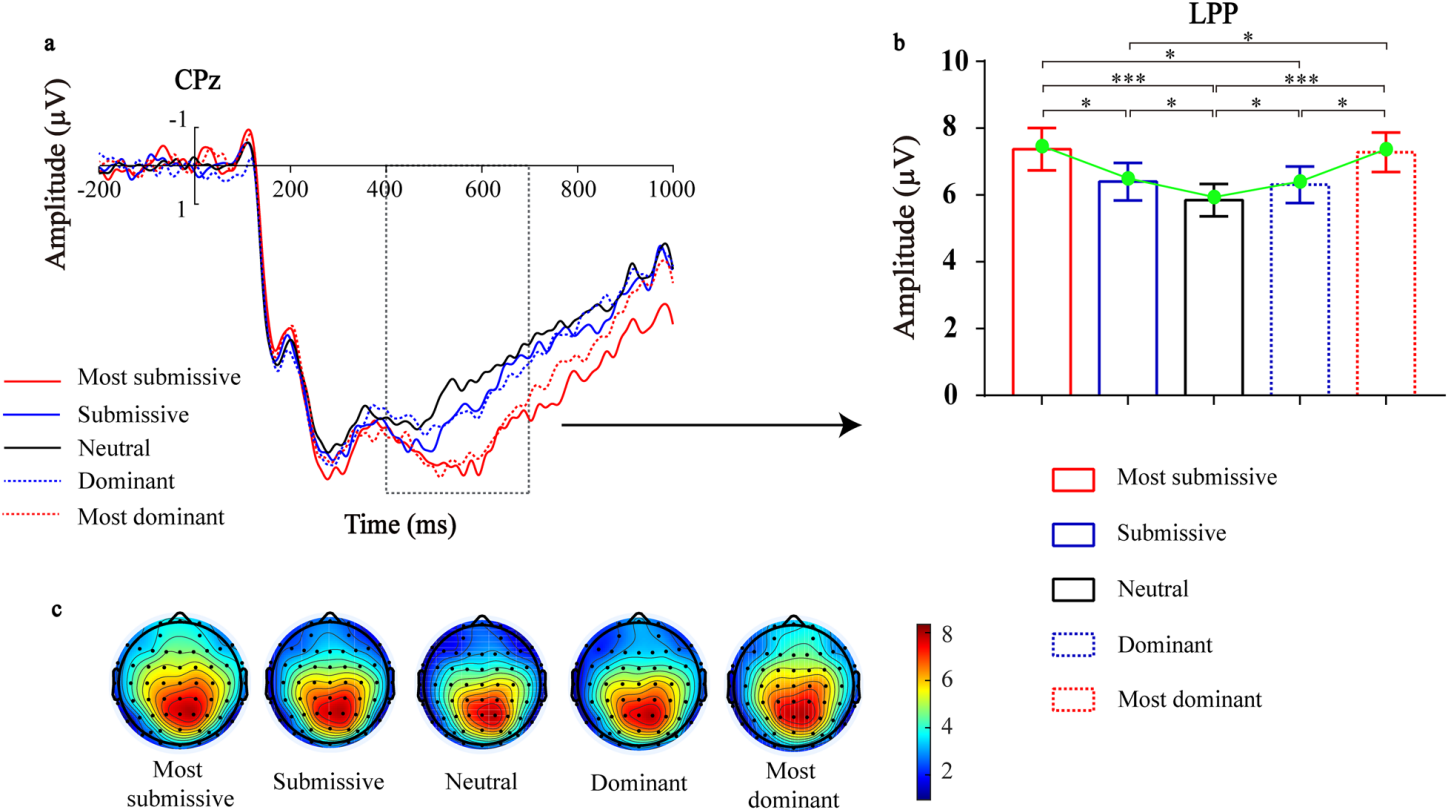
The LPP related to facial dominance evaluation. (**A**) The LPP over the centro-parietal region (CPz) as a function of dominance levels (− 3 SD, − 2 SD, 0 SD, + 2 SD, + 3 SD). (**B**) Bar plots illustrate the effect of dominance levels on the amplitude of the LPP over the centro-parietal region (CPz). Error bars indicate SEM, (*p < 0.05, ***p < 0.001). (**C**) Topographical voltage distributions within 400–700 ms centered on the peak of LPP elicited by faces with different levels of dominance. Positive isopotential lines are in red and negative isopotential lines are in blue.

The repeated-measures ANOVA performed on the mean amplitude of LPP revealed a significant main effect of dominance level (*F* (1, 26) = 6.284, *p* < 0.001, *η*^2^_*p*_ = 0.195). Post-hoc tests showed that the LPP amplitude was significantly greater both for the highest level of dominant faces and the lowest level of submissive faces than for all other levels (all *ps* < 0.05), and the LPP amplitude was significantly larger for the intermediate level of dominant faces and the intermediate level of submissive faces than for the neutral dominance faces (all *ps* < 0.05) (Fig. 3). Furthermore, there was a significant main effect of region (*F*(1, 26) = 9.055, *p* < 0.001, *η*^2^_*p*_ = 0.258). The post-hoc test showed that the LPP amplitude was significantly lower in the central region than in the centro-parietal (t = 5.458, *p* < 0.001) and parietal regions (t = 3.005, *p* = 0.006). In addition, there was also a significant main effect of laterality (*F*(1, 26) = 12.988, *p* < 0.001, *η*^2^_*p*_ = 0.333). Post-hoc tests further revealed a larger amplitude of the LPP in the electrodes on the right than that at the midline (t = 2.781, *p* = 0.01) and the left (t = 4.377, *p* < 0.001), and a greater amplitude of the LPP in the electrodes at the midline than that on the left (*p* = 0.009). No other significant effect was found.

## Discussion

The goal of the present study was to investigate the electrophysiological responses elicited by dominance features from human faces. Unlike previous EEG studies^24–27,29,31^, where dominance hierarchies conveyed by faces were built through competitive interactions, we relied on a variation of features of emotionally neutral faces signaling different levels of dominance. We focused on the N170 and LPP components, previously found to be modulated by facial dominance during face processing^24,26–29,31^. In contrast to previous ERP findings that linked professional rank to body posture, or, presenting faces along with dominance-conveying symbols^26,27,31^, we did not observe modulations by dominance hierarchy over the N170 component during face processing. However, we did find a modulation by dominance hierarchy over the LPP component during face processing. These findings show that participants’ processing of dominance features from human faces occurs during late evaluations of faces on social dimensions. Specifically, the highest level of dominant faces (+ 3 SD) and the lowest level of submissive faces (− 3 SD) elicited higher amplitudes compared with faces with the neutral dominance level (0 SD). Moreover, the intermediate level of dominant faces (+ 2 SD) and the intermediate level of submissive faces (− 2 SD) elicited higher amplitudes than the face with the neutral dominance level (0 SD).

### The role of dominance hierarchy over the N170

In the present study, we did not observe modulation of the N170 by different levels of dominance from human faces. Regarding the possible role of dominance in the early stage of face processing, as indexed by the N170, there is a discrepancy in the current literature. Specifically, the modulation by facial dominance on the N170 has consistently been reported in EEG studies where facial dominance was conveyed through perceptual cues that were either postures^31^ or faces combined with dominance-conveying symbols (e.g., different numbers of stars)^26,27^. In contrast, no N170 modulation was reported in studies in which facial dominance was learned either via the direct experience of competitive interactions^24^ or via presenting occupational labels followed by human faces^25^. Such discrepancy in the N170 findings has been argued to be accounted for by methodological differences in manipulating facial dominance^25^. Thus, in the situations where facial dominance was manipulated through either learning from the outcome of direct interactions or by associating professional dominance with the faces, one may deduce that inferring dominance hierarchies from the faces would not need to rely on perceptual identification of explicit dominance-conveying cues (e.g., stars). Instead, it might rely primarily upon memories of dominance hierarchies during the learning phase or knowledge of professional dominance. Consequently, participants’ facial dominance processing would occur during the cognitive assessment of faces and category-related information, but would not appear during the encoding of human faces. Here, it is noted that in those studies that show modulation of inferring dominance levels from human faces on the N170^26,27,31^, there exists a potential confounding effect induced by dominance-conveying symbols along with the faces or faces with dominant or submissive facial expressions on facial dominance judgments. This makes it impossible to determine whether the observed dominance level over N170 indeed reflects participants’ evaluation of facial dominance information or their assessment of dominance-conveying symbols along with the faces. To elucidate this issue, one possible solution could be to manipulate facial dominance features to eliminate such confounding effects. Following this perspective, the present approach manipulates facial dominance as features related to physical strength/weakness. This allows us to directly address the issue of whether facial dominance evaluation occurs during the early stage of face processing, and using this approach, no effect of facial dominance hierarchies on the N170 was found.

### The role of dominance hierarchy over the LPP

Consistent with previous research^24,25^, we found an influence of dominance hierarchy over the LPP. Specifically, we found that the highest level of dominant faces produced larger LPP amplitude than the intermediate level and dominance-neutral faces, and the intermediate level of dominant faces yielded greater LPP amplitude than dominance-neutral faces. Considering that the LPP has been proposed to reflect the processing of motivationally relevant stimuli such as sexual stimuli, money, and appetitive stimuli^35–37^ and facial evaluations on social dimensions^38^, our results seem to suggest that facial cues used to judge individuals’ dominance can represent motivationally relevant stimuli. Similar LPP modulations were also reported in recent electrophysiological studies in which facial dominance was built based on previous competitive experience^24,26,28^ or wealth^25,29^. Drawing on this literature, the findings described above provide additional evidence supporting the claim that faces with high dominance are socially and evolutionarily more desirable and high-dominance information conveyed by faces is thus more attractive and salient. Similarly, since a submissive face provides a meaningful social signal that an individual’s position in the dominance hierarchy is inferior, and since an individual’s position in the dominance hierarchy corresponds with their opportunities to prosper and succeed, the inferiority in dominance hierarchies is also of critical importance. Therefore, inferiority conveyed by subordinate faces in dominance hierarchies should also be cognitively and emotionally more salient and significant than dominance-neutral faces. Consistent with this view, a recent study^24^ reported that individuals bias the allocation of attentional resources to dominance and submissive information conveyed by human faces in a similar way. Moreover, the fusiform gyrus and in middle occipital gyrus have been found to show a quadratic response to dominance, with greater responses to extremes (submissive or dominant faces) than to moderate dominance^39^. Therefore, it is rational to speculate that dominant and submissive faces centering on dominance-neutral faces should be related to increased LPP amplitude for both dominant and submissive faces compared to dominance-neutral faces to a similar extent. Our findings on the modulation of the LPP by submissive faces support this argument by showing that the lowest level of submissive faces produced larger LPP amplitude than the intermediate level and dominance-neutral faces, and the intermediate level of submissive faces yielded greater LPP amplitude than dominance-neutral faces. Taken together, these findings add to the evidence supporting the view that the LPP reflects the recruitment of attentional and motivational systems dedicated to evaluating highly significant social stimuli.

### Potential limitations

Despite the promising findings in the present study, we should consider several potential limitations. First, it included a relatively small sample, possibly tempering the strength of our conclusions. Replications with larger samples would be welcome. Second, we did not record the gaze of participants. It remains to be determined whether facial dominance is encoded as whole-face information or whether it results primarily from the eye parameters covarying with facial dominance^40^. Taking this into account in future studies would further help to shed additional light on the neurocognitive subprocesses of facial dominance evaluation.

## Conclusion

In the present study, we varied the physical features of emotionally neutral faces in terms of physical strength/weakness to evoke dominance hierarchies to characterize the neurocognitive subprocesses of facial dominance evaluation. We did not observe modulations of the N170 component by dominance hierarchy during face processing. In contrast, we found modulations by dominance hierarchy over the LPP component during face processing. Furthermore, we revealed that the highest level of dominant faces and the lowest level of submissive faces elicited higher LPP amplitudes compared with faces with other dominance levels, and the intermediate level of dominant and submissive faces elicited higher LPP amplitudes than dominance-neutral faces. This supports that the LPP reflects the recruitment of attentional and motivational systems dedicated to evaluating more significant social stimuli. In this way, our results advance our understanding of the nature of neurocognitive subprocesses of facial dominance evaluation and thus have important implications for understanding how facial dominance exerts a temporally dynamic modulation of face processing.

## Methods

### Participants

Twenty-seven participants (15 females, *M*_age_ = 23.48 years, *SE* = 0.50), recruited from the University of Nanjing psychology participant pool, completed the study. We performed a prior power analysis to determine the sample size using G*Power Version 3.1^41^. Our power analysis showed that at least 21 participants would provide greater than 80% power (α = 0.05) to detect an effect of medium size (*η*^2^_*p*_ = 0.05) in our experimental design. Based on previous EEG studies focusing on facial dominance evaluation^24,25^, medium effects are what one can expect in this area of research. In reality, we recruited 27 participants to ensure the present study's robustness. All participants had normal or corrected-to-normal vision and were naive with regard to the purpose of the experiment. All participants gave written informed consent prior to participation. This study was approved by the Ethical Review Board of the University of Nanjing and conducted in accordance with the Declaration of Helsinki.

### Stimuli and experimental design

**Figure 4:**
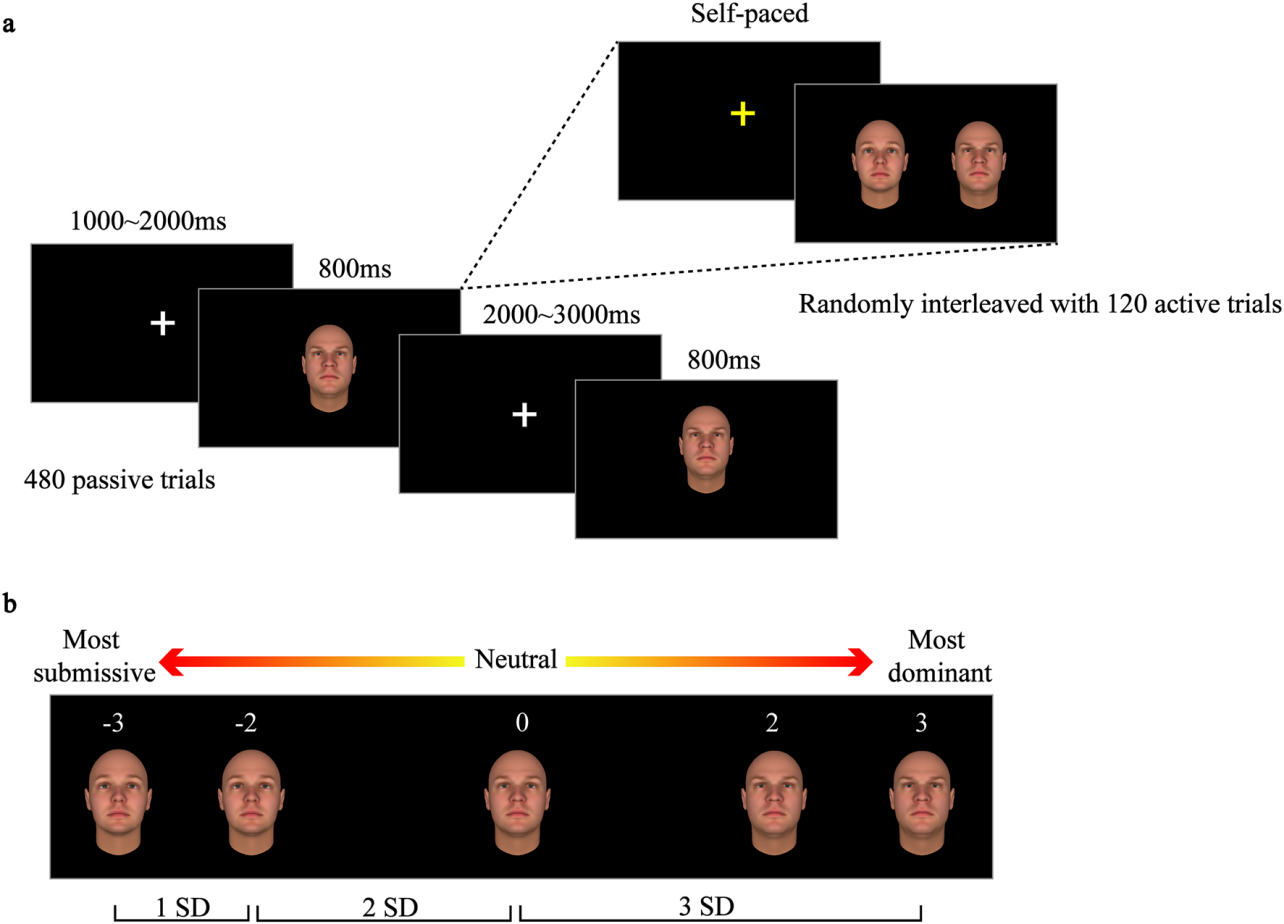
Experimental paradigm. (**A**) Participants were passively presented with computer-generated faces of 24 different face identities each of which could be morphed to vary along the social dominance dimension in five dominance conditions (− 3 SD, − 2 SD, 0 SD, + 2 SD, + 3 SD). Passive trials were repeated and were randomly interleaved with active trials in which participants had to perform a perceptual judgment to identify the more dominant face in a pair of faces. During active trials, pairs of faces were presented and their distance from the dominance-neutral face determined the level of difficulty to make that judgment (i.e., pairs with low distance constitute high-difficulty trials whereas pairs with high distance are low-difficulty trials). (**B**) Example of faces with variable dominance features. The face in the center is the dominance-neutral face whose facial features were exaggerated to decrease or increase its perceived dominance.

We used the existing database of the validated, computer-generated faces reported by previous studies in which emotionally neutral faces varied along the dominance dimension^33^. Twenty-five different face identities were available. Given that the social judgment of these faces in terms of dominance has been found to be universal across cultures and geographies^42^, the use of these faces should not introduce any confounding effect between face race and perceivers in our study, even though these faces were generated by using only Caucasian faces. For each face identity, faces differing maximally in dominance were created by moving a given identity’s neutral face along the dominance dimension ranging from − 3 to + 3 standard deviation (SD) in steps of 1 SD. This study used five faces of each identity corresponding to dominance levels − 3, − 2, 0, + 2, and + 3 SD. Participants were required to perform the dominance perception task, consisting of 480 passive trials randomly interleaved with 120 active trials (Fig. 4A). During the passive trials, a white fixation cross was presented at the center of the screen (1000–2000 ms) which was followed by a displayed face for 800 ms. In these passive trials, participants were required to view the faces and did not need to make any explicit responses. If the fixation cross was changed to yellow, this indicated that the following trial was an active trial. During active trials, a pair of faces of a given face identity with different dominance levels were presented and participants were instructed to indicate as quickly and accurately as possible which face was more dominant, by pressing either the left or right response button. Three levels of difficulty were defined: low difficulty, an interval of 3 SD between the two faces (e.g., level − 1 SD and level + 2 SD); medium difficulty, an interval of 2 SD between the two faces (e.g., level − 1 SD and level + 1 SD), and high difficulty, an interval of 1 SD between the two faces (e.g., level − 1 SD and the neutral face (level 0) (Fig. 4B). Stimuli were presented using the Presentation software 20.1.

Before the formal experiment, participants were asked to engage in a practice block in which participants were required to infer dominance levels of 5 faces of a given face identity to confirm that participants can accurately discriminate different levels of facial dominance related to physical strength/weakness. This face identity used in the practice block was not used in the formal experiment.

### EEG data recordings and analysis

We employed an EEG data recording procedure similar to that described in our previous studies^43–46^. Specifically, participants were seated in a soundproof, dimly lit room. Resting-state EEG data were recorded (SynAmps amplifier, NeuroScan) with a quick cap carrying 64 Ag/AgCl electrodes placed at standard locations covering the whole scalp (the extended international 10–20 system). The reference electrode was attached to the left mastoid (M1), and the ground electrode was placed on the forehead. The vertical electrooculogram (VEOG) was recorded with electrodes placed above and below the left eye. The horizontal electrooculogram (HEOG) was recorded using electrodes placed beside the two eyes. Impedance was kept below 5 kΩ. Electrophysiological data were continuously recorded with a bandwidth of 0.05–100 Hz and sampled at a rate of 1000 Hz. It was possible to observe participants via a video monitoring system.

Consistent with our recent work^44,45^, offline data analysis was performed using EEGLAB^47^ and ERPLAB^48^. The raw data were first re-referenced to linked mastoid (M1 and M2) and were filtered with a bandpass of 0.05–35 Hz and a notch (50 Hz) filter. Then, an independent component analysis (ICA) based artifact correction was conducted by using the ICA function of EEGLAB^47,49^. Independent components with topographies representing saccades blinks and heart rates were thus removed according to published guidelines^50^. The resultant EEG data were subsequently epoched from 200 ms pre-stimulus to 1400 ms post-stimulus. Baseline correction was applied from − 200 to 0 ms before stimulus onset. In order to remove movement artifacts, epochs were rejected when fluctuations in potential values exceeded ± 100 μV in any channels except the EOG channel. The ERPs evoked by five dominance levels (− 3, − 2, 0, + 2, and + 3 SD) were thus calculated by averaging individual artifact-free trials in each participant. Finally, the grand-averaged ERPs were computed and averaged for those levels.

### Statistical analysis

For the behavioral data during active trials, statistical significance was assessed with a one-way repeated analysis of variance (ANOVA) with difficulty (high, medium, and low) as a within-participant factor on both reaction times (RTs) and accuracy (the percentage success of selecting the more dominant-looking face).

Electrophysiological data were analyzed according to the topographical distribution of grand averaged ERP activity as well as according to the methods of previous work^24,25,31^. Our statistical analysis of ERP involved two ERP components that are found to be associated with facial dominance processing: the N170 and the LPP. The peak latency and mean amplitude of N170 were measured on temporoparietal sites (electrodes: P7 and P8). Peak latencies of this early ERP component were defined as the latency of the greatest negative deflection in a 140–180 ms time window. Its mean amplitude was quantified as the mean voltage across the 50 ms window that centered on its grand-average peak latency. A two-way ANOVA was conducted for this early ERP component, with dominance hierarchies and electrode sites as within-participant factors. Finally, the mean amplitude of this late ERP component over the central, centro-parietal, and parietal regions (C1, Cz, C2, CP1, CPz, CP2, P1, Pz, P2) was analyzed in a 400–700 ms time window. For this time window, a three-way ANOVA was conducted, with dominance levels, hemisphere (left, midline, and right), and region (central, centro-parietal and parietal) as within-participant factors.

All data were analyzed using IBM SPSS 21.0 (https://www.ibm.com/products/spss-statistics). Statistical comparisons were made at p-values of *p* < 0.05, with the Greenhouse–Geisser correction when violations of sphericity occurred.

## Data Availability

The datasets generated during and/or analyzed during the current study are available from the corresponding author upon reasonable request.
